# The effect of repetitive exposure to intravenous anesthetic agents on the immunity in mice

**DOI:** 10.7150/ijms.41899

**Published:** 2020-02-04

**Authors:** Hyun Jun Park, Liyun Piao, Eun-Hye Seo, Seung Hyun Lee, Seong-Hyop Kim

**Affiliations:** 1 Department of Infection and Immunology, Konkuk University School of Medicine, Seoul, Korea.; 2 BK21 plus, Department of Cellular and Molecular Medicine, Konkuk University School of Medicine, Seoul, Korea.; 3 Department of Microbiology, Konkuk University School of Medicine, Seoul, Korea.; 4 Department of Medicine, Institute of Biomedical Science and Technology, Konkuk University School of Medicine, Seoul, Korea.; 5 Department of Anesthesiology and Pain medicine, Konkuk University Medical Center, Konkuk University School of Medicine, Seoul, Korea.

**Keywords:** intravenous anesthetic agent, dexmedetomidine, midazolam, propofol, immunity

## Abstract

**Introduction**: This study was designed to assess the effect of repetitive exposure to intravenous anesthetic agents on the immunity in mice.

**Materials and Methods**: The mice were divided into six groups: three intravenous anesthetic agents groups (dexmedetomidine, midazolam and propofol groups), and three corresponding control groups (C_D_, C_M_, and C_P_ groups). The intravenous injections were administered once per day for 5 days. The immunity of mice was checked after the last intravenous injection. Histopathology and immunochemistry of liver and kidneys were evaluated. Cytokine levels in the blood was also checked. vs. evaluated with cytokine levels in the blood.

**Results**: Cluster of differentiation (CD)4^+^ T cells were significantly less expressed in dexmedetomidine and propofol groups, compared with the corresponding control groups [34.08 ± 5.63% in the dexmedetomidine group vs. 59.74 ± 8.64% in the C_D_ group, *p < 0.05*; 25.28 ± 7.28% in the propofol group vs. 61.12 ± 2.70% in the C_p_ group, *p < 0.05*]. Apoptosis of CD4^+^ T cells was increased significantly in dexmedetomidine and propofol groups, compared with the corresponding control groups. Histopathological findings of liver and kidneys did not show any specific differences of any of three intravenous anesthetic agents groups with their corresponding control groups, although immunohistochemical examination indicated significantly lower expression of Toll-like receptor-4 from liver and kidneys in dexmedetomidine and propofol groups. The cytokine levels were not different between the groups.

**Conclusion**: Repetitive exposure to dexmedetomidine and propofol reduced the expression of CD4^+^ T cells but did not induce any significant liver or kidney injuries.

## Introduction

Sedation is widely performed in clinical situations to facilitate diagnostic and therapeutic procedures. The most commonly used intravenous anesthetic agents for sedation are dexmedetomidine, midazolam, and propofol. The action of sedation is expressed via α_2_-adrenergic receptors for dexmedetomidine and γ-aminobutyric acid (GABA) receptors for midazolam and propofol 1-4. Both α_2_-adrenergic receptors 5, 6 and GABA receptors 7-9 modulate neurotransmitters and influence the immune system 10, 11. Repeated sedation for a short time is sometimes performed on the basis of patient status— for example, pediatric patients. Depending on the intravenous anesthetic agents used, different immunity patterns would be expected. However, this topic has not yet been investigated.

We hypothesized that repeated exposure to anesthetic agents might affect the immunity differently, depending on the intravenous anesthetic agent administered. This study was designed to assess the effect of 5 days of repetitive exposure to intravenous anesthetic agents, including dexmedetomidine, midazolam, and propofol, on the immunity in mice.

## Materials and Methods

These experiments were approved by the Institutional Animal Care and Use Committee (IACUC) of the Konkuk University (KU16126) and conducted at the Konkuk University Laboratory Animal Research Center. The experiments were performed by following the IACUC guidelines for studying laboratory animals.

The data used to support the findings of the study are available from the corresponding author (Seong-Hyop Kim, yshkim75@daum.net) upon request.

Six-week-old male BALB/c mice (weight 20 g) were used for the experiment. The animals were quarantined for 2 weeks to confirm that they were pathogen-free. The mice were divided into six groups: three intravenous anesthetic agents groups (dexmedetomidine, midazolam, and propofol groups) and three corresponding control groups (C_D_ group for dexmedetomidine, C_M_ for midazolam, and C_P_ for propofol).

### Anesthetic method

The mice were placed in a clean dry cage without bedding before anesthesia, to avoid ingestion or inhalation during the procedure. The mice were maintained in room air at 25°C throughout the procedure to prevent hypothermia. Dexmedetomidine (0.4 mg/kg; Dexmedine inj., Hana Pharm, Seoul, Korea) midazolam (50 mg/kg Vascam inj.; Hana Pharm), propofol (26 mg/kg Anepol inj.; Hana Pharm) and normal saline for the corresponding control groups were intravenously injected through the tail vein 12-14. One milliliter was administered to animals in all groups. The mice were laid in the supine position in a V-shaped trough. The hypnotic response was confirmed by loss of the righting reflex (LORR). The LORR was defined as the inability of the mice to right themselves. The induction time from injection of the study drug to LORR was recorded. When the mice righted themselves after LORR, they were laid in the supine position again. Recovery of the righting reflex was defined as the ability of the mice to right themselves twice within 60 s. LORR duration was defined as the time interval between loss and recovery of the righting response. The sedated mice were carefully observed to check for any complications. A radiant heating lamp was applied to maintain body temperature during the procedure. The intravenous injections were administered once per day for 5 days. All procedures were performed by the same investigator at the same time. Respiration rate was observed during all procedures. Body temperature was monitored with a rectal thermometer and maintained above 36°C. The mice were euthanized by cervical dislocation immediately after the last intravenous injection. Immune cells undergoing apoptosis in the blood were checked by flow cytometry to evaluate the immunity. Histopathology and immunochemistry of the liver and kidney were also performed. We checked blood cytokine levels by enzyme-linked immunosorbent assay (ELISA).

### Peripheral blood mononuclear cells (PBMCs)

After excising the spleen, the diaphragm was incised and the heart exposed. Blood samples were obtained from heart punctures using heparin-coated 2 ml syringes and collected in 1.5 ml Eppendorf tubes (Sigma Aldrich, St. Louis, MO, USA). The whole blood was centrifuged at 3,000 rpm for 5 min to separate the serum. The serum samples were isolated and saved in another 1.5 ml tube at -20°C to check the cytokine levels. The remaining blood was diluted in phosphate-buffered saline (PBS) to prevent coagulation, and PBMCs were separated from the remaining blood by Ficoll density-gradient centrifugation at 2,400 rpm for 20 min at room temperature. The PBMCs were washed in PBS and aliquoted into two 5 ml round-bottom tubes. They were then washed in fluorescence-activated cell sorting (FACS) buffer.

### Immunofluorescence staining of immune cells

To confirm innate and adaptive immune systems, respectively, neutrophils for innate immune system, providing a rapid response, and cluster of differentiation (CD)4^+^ T cells, CD8^+^ T cell and CD4^+^CD25^+^ T cell for adaptive immune response, providing a slow but highly specific response, were chosen. They undergoing apoptosis were evaluated using isolated PBMCs.

The pellet in the 5 ml round-bottom tube with isolated PBMCs was stained with the monoclonal antibody peridinin-chlorophyll (PerCP) CD11b (Biolegend, Dedham, MA, USA), Fluorescein isothiocyanate (FITC) Ly6G and allophycocyanin (APC) Ly6C (Biolegend) were used to check the neutrophils. The pellet in the 5 ml round-bottom tube with isolated PBMCs was stained with PerCP CD25 (Biolegend) and APC CD4 (Biolegend) monoclonal antibodies to check the CD4^+^ and CD4^+^CD25^+^ T cells; and with PerCP CD3 (Biolegend) and APC CD8 (Biolegend) monoclonal antibodies to check the CD8^+^ T cells. The cells were incubated for 30 min at room temperature in the dark. The cells were washed in 500 μl of cell staining buffer (Biolegend). To check for apoptosis, 300μl of binding buffer (Biolegend) and FITC Annexin V (Biolegend) were added and incubated for 30 min in the dark at room temperature. After the incubation, the population of immune cells undergoing apoptosis was measured and analyzed with the FACS Accuri C6 flow cytometer (BD Biosciences, Seoul, Korea).

### Tissue preparation for histopathology and immunohistochemistry

The order of the procedures for histopathology and immunohistochemistry was as follows: 1) tissue preparation, 2) fixation, 3) dehydration, 4) embedding, and 5) staining.

The liver and kidneys were obtained from the mice and fixed overnight at 25°C in a 4% paraformaldehyde solution (Biosesang, Seoul, Korea). The fixed liver was cut through the caudate and left lateral lobes. The fixed kidneys were transected. The dissected organs underwent tissue processing using a tissue processor (TP1020^®^; Leica Biosystems, Lincolnshire, IL, USA). The dissected organs were dehydrated through a series of graded ethanol baths to displace water and then infiltrated with wax. The organs were embedded in paraffin using an embedding center (EG1150^®^; Leica Biosystems). Tissue sections were cut to 4-μm thickness using a microtome (Leica Biosystems) and mounted on poly-l-lysine coated microscopic slides (Mutokagaku, Tokyo, Japan).

### Histopathological examination

The histopathological examination was conducted with hematoxylin and eosin-stained slides. The sections were deparaffinized and rehydrated. Then, the slides were stained with hematoxylin (Sigma-Aldrich) for 3 min in the dark. After staining, the slides were washed in tap water. The slides were then stained with eosin (Sigma-Aldrich) for 30 sec and rewashed in tap water. The slides were dehydrated and cover-slipped using mounting medium and images were obtained under a microscope.

The liver injury score was defined by sinusoidal congestion, hepatocyte necrosis, and ballooning degeneration and scored from 0 to 4: 0, no necrosis, congestion or ballooning; 1, minimal congestion, single-cell necrosis or ballooning; 2, congestion, ballooning degeneration or lobular necrosis < 30%; 3, moderate congestion, ballooning degeneration or lobular necrosis < 60%; 4, severe congestion, ballooning degeneration or lobular necrosis > 60%.

The renal injury score was defined by the degree of tubular cell damage and ranged from 0 to 4: 0, no damage; 1, unicellular or patchy isolated necrosis; 2, tubular necrosis < 25%; 3, tubular necrosis of 25-50%; 4, > 50% tubular necrosis and presence of infarcted tissue.

### Immunohistochemical examination

Toll-like receptor-4 (TLR4) is a transmembrane protein, and its activation leads to an intracellular signaling pathway and production of inflammatory cytokines, responsible for activating the innate immune system. Therefore, TLR4 was used to check the immunity in the tissues. The slides were deparaffinized and rehydrated. To avoid non-specific binding of antibody, the slices were incubated in blocking solution (Vector Laboratories, Burlingame, CA, USA) for 1 h and reacted with TLR4 rabbit polyclonal antibody (Abcam, Cambridge, UK) at a 1:100 dilution overnight at 4°C. The slides were then washed in PBS, and the sections were incubated for 1 h with biotinylated secondary goat anti-rabbit IgG (Abcam). After the incubation, the ABC Reagent (Vector Laboratories) was applied to react with the biotinylated antibody for 1 h at 25°C and attached with 3,3`-diaminobenzidine reagent (Vector Laboratories). The slides were stained with hematoxylin as a counterstain, dehydrated and cover-slipped using mounting medium (Vector Laboratories). Images were obtained under a microscope (Nikon, Tokyo, Japan). TLR4 intensity was quantified using Image J software (NIH, Bethesda, MD, USA).

### Cytokines

The levels of interleukin (IL)-2, interferon (IFN)-γ, tumor necrosis factor (TNF)-α, and transforming growth factor (TGF)-β were checked in serum by ELISAs.

### Statistical analysis

“Resource equation method” was used to determine sample size because it was impossible to assume the effect size or no previous published studies for power analysis. With the formula for the resource equation method (E = Total number of animals - Total number of groups, any sample size, which keeps E between 10 and 20, should be considered to be adequate.), total number of animals between 12 and 22 for an intravenous anesthetic agent (between 36 and 66 for the three intravenous anesthetic agent including the corresponding control group) was adequate for sample size determination. Differences between groups were analyzed with the Mann-Whitney U test using GraphPad Prism software (ver. 5.01; GraphPad Software, La Jolla, CA, USA). Data are presented as means ± standard deviation [median (25-75%)]. A *p*-value < 0.05 was considered significant.

## Results

A total of 36 mice were used for the experiments and evenly allocated to the six groups. No complications occurred.

The induction time and duration of LORR were longer in the dexmedetomidine group than in the other intravenous anesthetic groups (Table 1). The propofol group had the shortest LORR induction time and duration (Table 1).

**Figure 1:**
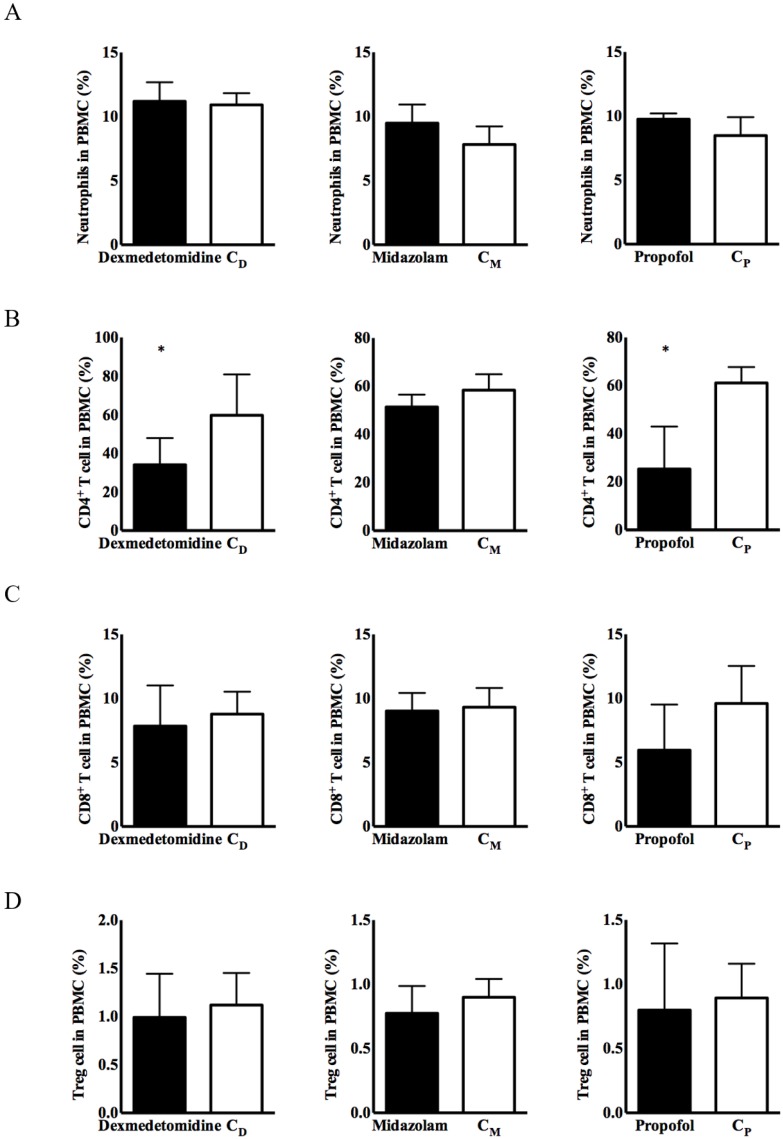
** Expression of immune cells in peripheral blood mononuclear cells (PBMCs). (A)** Neutrophils, **(B)** cluster of differentiation (CD)4^+^ T cells, **(C)** CD8^+^ T cells, and **(D)** CD4^+^CD25^+^ T cells. Abbreviations: C_D_ group, corresponding control group for dexmedetomidine; C_M_ group, corresponding control group for midazolam; C_p_ group, corresponding control group for propofol. ^*^p < 0.05 compared with corresponding control group.

No significant differences in the expression of immune cells were observed, except for CD4^+^ T cells, in any of the three intravenous anesthetic agents groups or the corresponding control groups (Figure 1). CD4^+^ T cells from PBMCs had significantly lower expression in the dexmedetomidine and propofol groups versus the corresponding control groups [34.08 ± 5.63% in the dexmedetomidine group vs. 59.74 ± 8.64% in the C_D_ group, *p < 0.05*; 25.28 ± 7.28% in the propofol group vs. 61.12 ± 2.70% in the C_p_ group, *p < 0.05*] (Figure 1).

**Figure 2:**
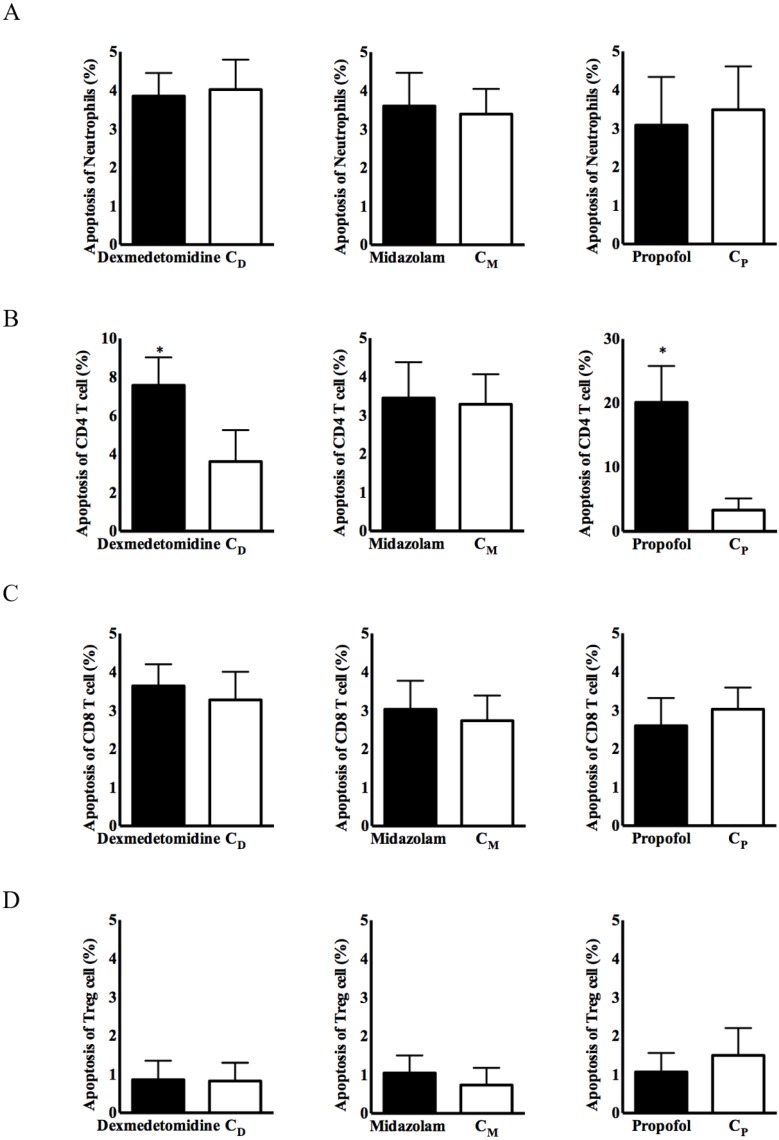
** Apoptosis of immune cells in PBMCs. (A)** Neutrophils, **(B)** CD4^+^ T cells, **(C)** CD8^+^ T cells, and **(D)** CD4^+^CD25^+^ T cells. Abbreviations: C_D_ group, corresponding control group for dexmedetomidine; C_M_ group, corresponding control group for midazolam; C_p_ group, corresponding control group for propofol; CD, cluster of differentiation. ^*^p < 0.05 compared with corresponding control group.

No significant group difference in immune cell apoptosis was observed, except for CD4^+^ T cells, in any of the three intravenous anesthetic agent groups and the corresponding control groups (Figure 2). Apoptosis of CD4^+^ T cells from PBMCs was significantly more frequent in the dexmedetomidine and propofol groups, compared with the corresponding control groups [8.15 ± 3.74% in the dexmedetomidine group vs. 3.62 ± 2.19% in the C_D_ group, *p < 0.05*; 23.77 ± 10.27% in the propofol group vs. 4.74 ± 3.32% in the C_p_ group, *p < 0.05*] (Figure 2).

**Figure 3:**
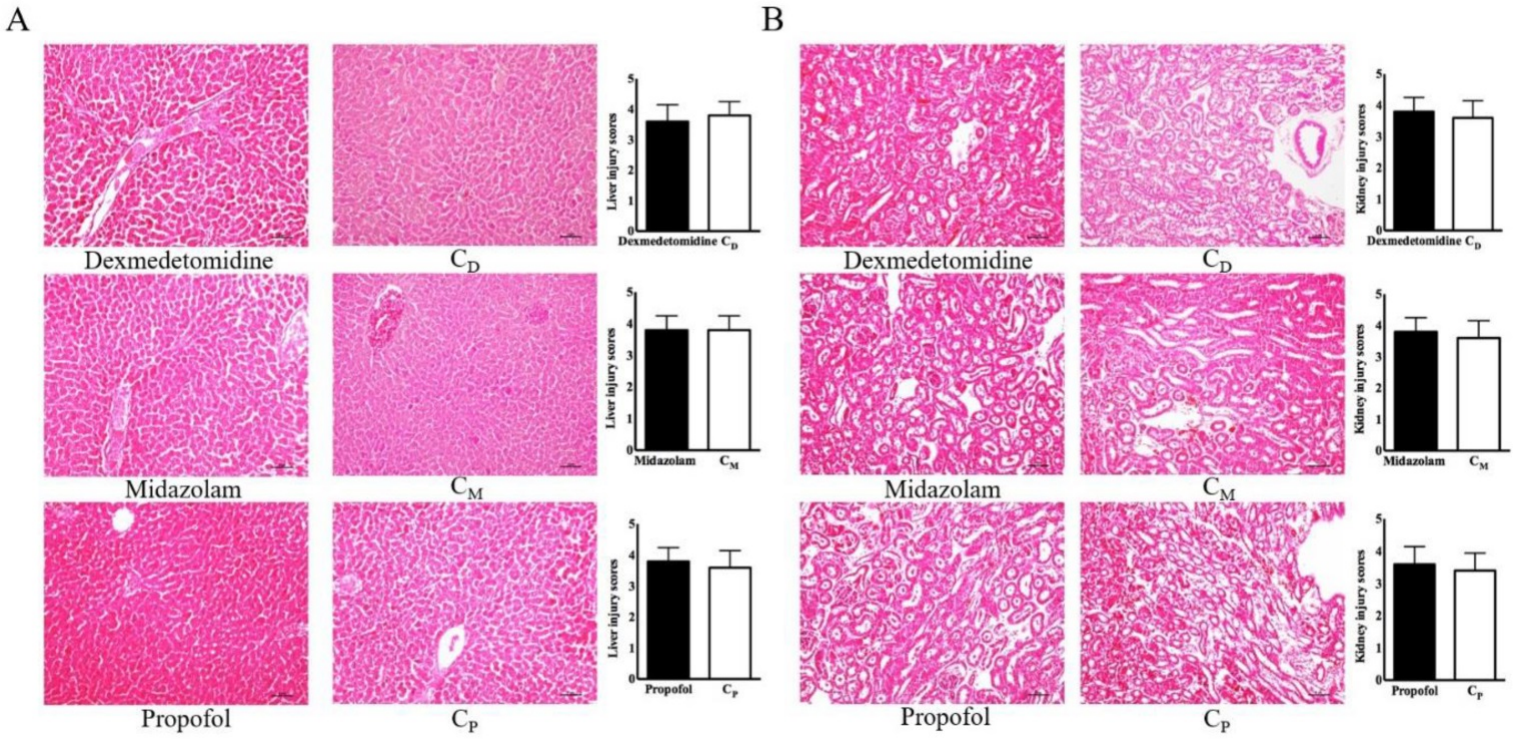
** Liver and kidney injury scores. (A)** Liver, **(B)** kidney. Abbreviations: C_D_ group, corresponding control group for dexmedetomidine; C_M_ group, corresponding control group for midazolam; C_p_ group, corresponding control group for propofol.

The histopathological findings in the liver and kidneys did not reveal any differences in the three intravenous anesthetic groups versus their corresponding control groups (Figure 3).

**Figure 4:**
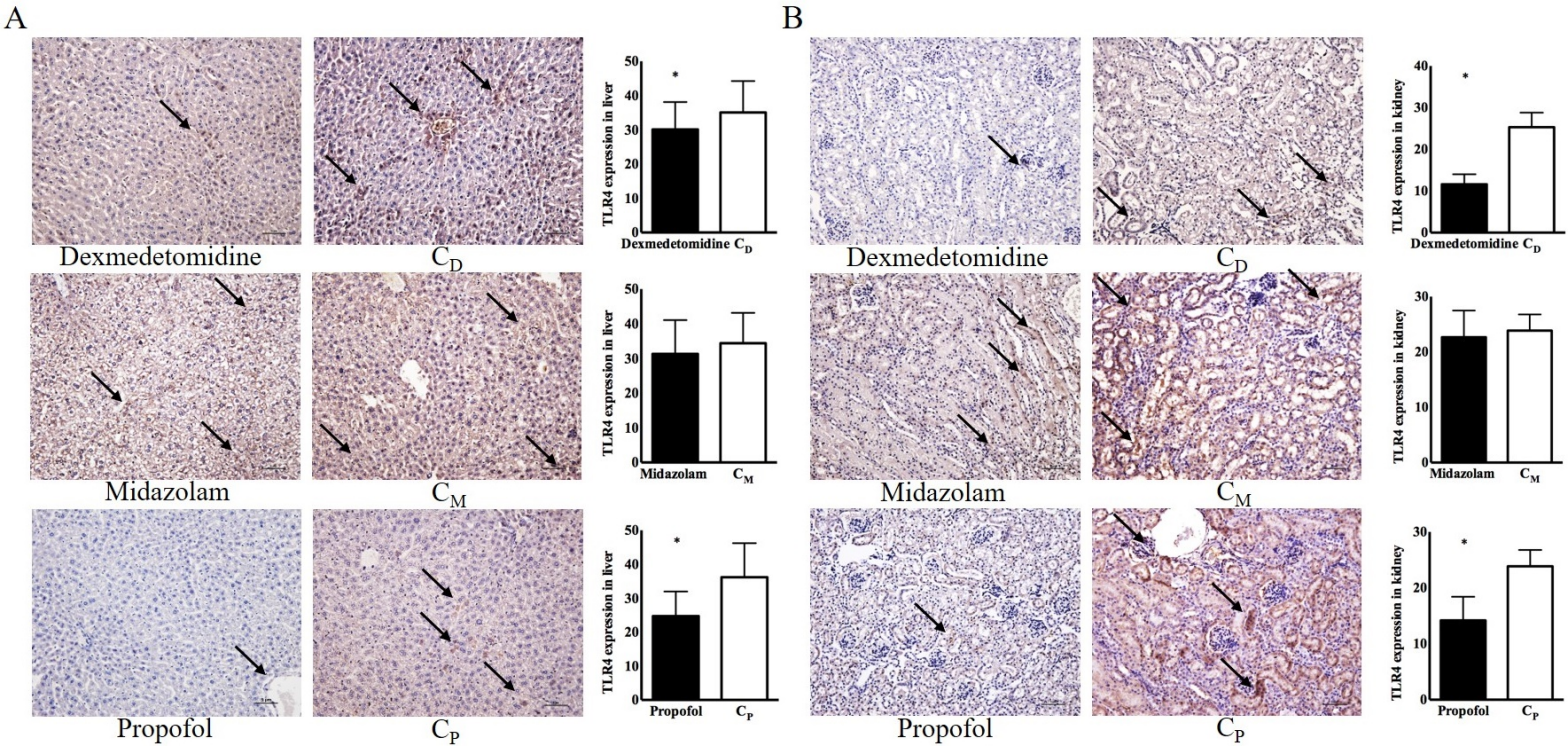
** Toll-like receptor-4 (TLR4) expression in liver and kidney. (A)** Liver, **(B)** kidney. Abbreviations: C_D_ group, corresponding control group for dexmedetomidine; C_M_ group, corresponding control group for midazolam; C_p_ group, corresponding control group for propofol. Black arrow indicates TLR-4 expression. ^*^p < 0.05 compared with corresponding control group.

Immunohistochemical examinations of the dexmedetomidine and propofol groups revealed significantly lower TLR4 expression in the liver [30.11 ± 3.57 in the dexmedetomidine group vs. 35.06 ± 4.11 in the C_D_ group, *p < 0.05*; 14.21 ± 1.88 in the propofol group vs. 23.87 ± 1.31 in the C_p_ group, *p < 0.05*] and kidneys [11.60 ± 1.08 in the dexmedetomidine group vs. 25.31 ± 1.56 in the C_D_ group, *p < 0.05*; 14.21 ± 1.88 in the propofol group vs. 23.87 ± 1.31 in the C_p_ group, *p < 0.05*] compared with the corresponding control groups (Figure 4).

The cytokine levels were not different in any of the three intravenous anesthetic groups and the corresponding control groups (Table 2).

## Discussion

This study showed that repetitive exposure to dexmedetomidine and propofol reduced the expression of CD4^+^ T cells in the serum, and the intensity of TLR4 expression in the liver and kidneys, with increased apoptosis in PBMCs. However, repetitive exposure to the three intravenous anesthetic agents did not result in any liver or kidney injuries with increasing cytokine levels.

There have been numerous studies for the effect of a specific intravenous anesthetic agent on the mechanism of immune response under a specific condition. However, the studies with a single injection or continuous infusion, not repetitive injection, of intravenous anesthetic agent have been conducted without any control group. The procedure under repetitive injection of intravenous anesthetic agent is very common in clinical situation. Therefore, the aim in the present study was to confirm the effect of repetitive exposure to intravenous anesthetic agent on immunity.

The significant differences in induction time and duration of LORR among the three intravenous anesthetic agents in the present study could be explained by their different pharmacokinetic and pharmacodynamic properties. Direct comparison among the three intravenous anesthetic agents was impossible. Therefore, we determined the maximal doses for sedation based on literature studies and compared them with the corresponding control groups 15-18. If a more objective surrogate for pharmacodynamics than LORR, such as electroencephalograph-based devices, was applied with the various doses of intravenous anesthetic agents used in the present study, a dose-response relationship could be derived and could give more information about immunity.

The present study showed different expression patterns of immune cells among intravenous anesthetic agents, suggesting that the agents have unique effects on the immunity. Repetitive exposure to dexmedetomidine and propofol reduced the expression of CD4^+^ T cells with increased apoptosis of PBMCs, indicating an immunosuppressive effect or protective effect against injury. This finding was supported by the lower expression of TLR4 in the liver and kidneys. However, no differences in cytokines were seen. Therefore, functional data for viable immune cells in the present study would be helpful. Wang et al. 19 reported that dexmedetomidine attenuated CD4^+^ T cells and restrained the phosphorylation of nuclear factor kappa-light-chain-enhancer of activated B cells (NF-κB), participating in the regulation of cytokine secretion in mice with hepatitis. Many studies have demonstrated that dexmedetomidine has a protective effect against injury through TLR4, with or without a reduction in cytokine levels 20-24. Propofol had a similar effect on immune cells and TLR4 expression. Although propofol increased the expression of immune cells compared with inhaled anesthetic agents, it significantly increased the apoptosis of lipopolysaccharide-treated mononuclear cells and lymphocytes 25-27. Many studies have supported a protective effect of propofol against injury through TLR4 28-32. The difference in results between propofol and midazolam in the present study was remarkable, although both are GABAergic drugs. Sedation from intravenous anesthetic agents can indirectly modulate the immunity, and intravenous anesthetic agents can directly affect the immunity. GABAergic drugs, including propofol and midazolam, are reported to have immune modulating effects 33. However, Yuki et al. 34 reported that propofol suppresses T cell proliferation, whereas midazolam does not. Midazolam also has a protective effect against injury; however, this is not due to direct modulation of immune cells, but rather a direct effect on target cells 35.

The protective effect of intravenous anesthetic agents with a short duration on the liver and kidneys would be associated with non-specific histopathologic findings 36-38.

Only CD4^+^ T cells among the immune cells in the present study were affected by dexmedetomidine and propofol, in association with time elapsed. The immune response usually occurs following particular steps. The innate immune response, including of neutrophils, protects against injury 40. The maximal activity of neutrophils occurs 1-3 days after an injury. Signals from the innate immune response activate the acquired immune response. The maximal activity of T cells occurs 5 days after injury. Most studies investigated the immunity within 5 days after a single injection of an intravenous anesthetic agent under specific conditions 39-42. However, the immunity in the present study was checked 5 days after daily, repeated administration of the intravenous anesthetic agents. Therefore, the results of the present study would be different from previous reports. Moreover, we did not induce any injury before repetitive administration.

In the present study, CD4^+^ T cell apoptosis was increased significantly in the dexmedetomidine and propofol groups compared with the corresponding control groups. Clinical trials of the effect of intravenous anesthetic agents on lymphocyte apoptosis are rare. Braz et al. 43 reported that patients under propofol anesthesia had lower levels of oxidized purines and apoptosis of helper T lymphocytes. Although evidence for the association between lymphocyte apoptosis and clinical impact is limited 44, the consequences from increased lymphocyte apoptosis are expected to decrease inflammatory factors and would lead to better clinical outcomes.

The immune response protects the host against pathogens. However, an excessive immune response can result in tissue injury. Therefore, a balance in the immune response is critical to maintain homeostasis. The choice of dexmedetomidine and propofol is helpful for patients requiring repetitive sedation who develop an excessive immune response, such as an autoimmune disease or hypersensitivity.

Several considerations in the present study should be discussed. First, emulsified propofol in dilution with normal saline, not pure propofol, was used in the present study. Also, the control group for propofol was tested with normal saline, not emulsifier. To check the effect of propofol on the immunity of mice, pure propofol in dilution with emulsifier and emulsifier as a control should be compared. However, propofol is used clinically with an emulsified formulation. The manufacturer of propofol recommends the use of normal saline or 5% dextrose water to dilute emulsified propofol 45. Moreover, dilution with normal saline is used to prevent pain from the injection of propofol 46. Second, the immunity after repetitive exposure to intravenous anesthetic agents would be transient and have less impact on the host. However, the impact in patients with immune disorders would be substantial and should be considered in situations involving repetitive exposure to intravenous anesthetic agents, although further evaluation is required to confirm this. Third, experiments on the effect of intravenous anesthetic agents on specific conditions such as immune-activated conditions, using lipopolysaccharide, support the results of the present study. However, the specific condition itself might influence the intravenous anesthetic agent-related immunity. Therefore, we performed our experiment in the absence of any specific disease to clarify the effect of the intravenous anesthetic agent itself on the immunity of the mice.

In conclusion, intravenous anesthetic agents for sedation should be chosen based on their pharmacokinetic and pharmacodynamic properties. Repetitive exposure to all three intravenous anesthetic agents in this study had no effect on liver and kidney injury, although dexmedetomidine and propofol reduced the expression of CD4^+^ T cells, and the intensity of TLR4 in liver and kidney with increased apoptosis in PBMCs.

## Figures and Tables

**Table 1:** Demographic data

	Dexmedetomidine group	C_D_ group	Midazolam group	C_M_ group	Propofol group	C_P_ group
**Total injected dose (mg/kg)**	0.4	-	50	-	26	-
**LORR induction time (sec)**	2310 ± 612 [2100 (1845-2400)]	-	24 ± 84 [0 (0-300)]	-	Immediately [0 (0-0)]	-
**LORR duration (sec)**	10470 ± 3864 [11700 (9960-12675)]	-	1116 ± 210 [870 (570-1200)]	-	300 ± 144 [240 (210-287.3)]	-

All values are means ± standard deviation [median (25%-75%)]. Abbreviations**:** C_D_ group, corresponding control group for the dexmedetomidine group; C_M_ group, corresponding control group for midazolam; C_p_ group, corresponding control group for propofol; LORR, loss of righting reflex.

**Table 2:** Serum cytokine levels

	Dexmedetomidine group	CD group	*p*-value	Midazolam group	CM group	*p*-value	Propofol group	CP group	*p*-value
**IL-2 (ng/ml)**	9.22 ± 1.34 [10.22 (7.67-11.30)]	10.76 ± 2.50 [11.25 (8.55-12.38)]	0.42	11.28 ± 2.18 [10.69 (10.40-13.87)]	10.68 ± 3.95 [9.55 (8.25-11.08)]	0.15	12.12 ± 3.40 [12.56 (9.87-14.24)]	11.94 ± 2.54 [12.36 (9.75-13.39)]	0.69
**IFN-γ (ng/ml)**	16.46 ± 3.20 [17.52 (14.75-18.72)]	15.29 ± 2.44 [16.28 (14.64-17.73)]	0.42	13.90 ± 2.83 [13.25 (11.75-14.73)]	14.88 ± 3.50 [13.56 (12.22-17.75)]	0.69	13.48 ± 2.58 [12.35 (12.29-15.75)]	13.90 ± 3.56 [13.56 (10.74-17.75)]	0.99
**TNF-α (pg/ml)**	23.11 ± 4.22 [23.26 (19.74-25.96)]	27.38 ± 5.22 [25.26 (24.00-30.62)]	0.06	25.37 ± 2.36 [24.65 (20.83-27.26)]	23.85 ± 3.94 [24.33 (19.91-27.15)]	0.84	27.22 ± 3.45 [29.48 (22.93-30.95)]	26.33 ± 4.85 [25.98 (22.63-30.87)]	0.99
**TGF-β (pg/ml)**	12.44 ± 2.34 [11.56 (10.30-13.58)]	13.42 ± 1.34 [13.69 (12.44-14.83)]	0.10	9.63 ± 1.52 [10.33 (8.23-10.69)]	11.83 ± 4.00 [10.11 (8.74-15.29)]	0.84	13.82 ± 1.74 [13.56 (11.70-14.45)]	12.34 ± 2.77 [11.56 (11.09-13.24)]	0.42

All values are means ± standard deviation [median (25%-75%)] Abbreviations: C_D_ group, corresponding control group for dexmedetomidine; C_M_ group, corresponding control group for midazolam; C_p_ group, corresponding control group for propofol; IL, interleukin; IFN, interferon; TNF, tumor necrosis factor, TGF, transforming growth factor.
